# Predicting Personalized Diets Based on Microbial Characteristics between Patients with Superficial Gastritis and Atrophic Gastritis

**DOI:** 10.3390/nu15224738

**Published:** 2023-11-09

**Authors:** Xiaoxiang Gao, Pingping Yin, Yilin Ren, Leilei Yu, Fengwei Tian, Jianxin Zhao, Wei Chen, Yuzheng Xue, Qixiao Zhai

**Affiliations:** 1 State Key Laboratory of Food Science and Resources, Jiangnan University, Wuxi 214122, China; mtmu2n@163.com (X.G.);; 2 School of Food Science and Technology, Jiangnan University, Wuxi 214122, China; 3 Department of Gastroenterology, Affiliated Hospital of Jiangnan University, Wuxi 214122, China; 4 National Engineering Research Center for Functional Food, Jiangnan University, Wuxi 214122, China

**Keywords:** atrophic gastritis, *H. pylori*, microbiome, dietary strategies, exogenous metabolites, microbiota-directed food

## Abstract

Background: gastritis is a common stomach disease with a high global incidence and can potentially develop into gastric cancer. The treatment of gastritis focuses on medication or diets based on national guidelines. However, the specific diet that can alleviate gastritis remains largely unknown. Methods: we propose a microbiota-directed dietary strategy that investigates potential food factors using microbial exogenous metabolites. Given the current lack of understanding of the repeatable characteristics of gastric microbiota, we conducted a meta-analysis to identify the features of gastric bacteria. Local samples were collected as validation cohorts. Furthermore, RevEcoR was employed to identify bacteria’s exogenous metabolites, and FooDB was used to retrieve foods that can target specific bacteria. Results: Bacteroides, Weissella, Actinomyces, Atopobium, Oribacterium, Peptostreptococcus, and Rothia were biomarkers between superficial gastritis (SG) and atrophic gastritis (AG) (AG_N) without *H. pylori* infection, whereas Bacillus, Actinomyces, Cutibacterium, Helicobacter, Novosphingobium, Pseudomonas, and Streptococcus were signatures between SG and AG (AG_P) with *H. pylori* infection. According to the exogenous metabolites, adenosyloobalamin, soybean, common wheat, dates, and barley were regarded as potential candidates for AG_N treatment, while gallate was regarded as a candidate for AG_P treatment. Conclusions: this study firstly profiled the gastric microbiota of AG and SG with or without *H. pylori* and provided a recommended diet for global AG according to exogenous metabolites.

## 1. Introduction

The majority of first-line food therapies are based on dietary guidelines, and these approaches do show effectiveness; for instance, the Mediterranean diet is known to alleviate hypertension [1,2]. However, due to substantial individual differences, the response rate of such dietary therapies is modest. In recent years, there has been significant attention directed towards the interaction between gut microbiota and complex dietary factors, as it ultimately enhances their bioavailability [3]. Understanding these interactions has become crucial for the advancement of personalized nutrition. In fact, microbiota-directed dietary interventions that specifically focus on certain bacteria have been verified, and specific dietary formulas can be designed to regulate malnourished microbiota in order to promote infant development [4]. The consumption of fiber snacks can stimulate the growth of *Bacteroides* and provide relief from obesity [5]. Therefore, the future holds great promise for the development of diets that target specific biomarkers and physiological characteristics.

Gastric carcinoma is the second most common cause of cancer-related mortality worldwide, with more than 1 million new patients diagnosed per year. Gastric carcinoma generally begins with the acquisition of *Helicobacter pylori* infection and nonmalignant lesions in the gastric body, such as atrophic gastritis (AG) [6]. *H. pylori* infection releases virulence factors that induce chronic inflammation and are risk factors for gastric carcinoma [7]. So far, the eradication of *H. pylori* is a major therapy suggested by the WHO international agency for gastric inflammation and cancer prevention. Although eradication effectively reduces the risk of gastric cancer, previous retrospective studies have reported that the trend of gastric carcinoma development and AG did not change [8].

It has been suggested that other factors, such as microbes, diet, and medicines, lead to stomach inflammation and the development of AG in people without *H. pylori* infection [9]. Among these, microbial communities lacking *H. pylori* play a crucial role in gastritis [7]. Gastric carcinoma progression was affected by non-*H. pylori* microbiota, as previously demonstrated in humanized mice. Transplanting gastric microbiota from patients with gastritis into germ-free mice reproduces the main histopathological features of precancerous lesions [10]. Until now, studies on *H. pylori*-related microbiota and gastritis-associated microbiota without *H. pylori* infection have been limited [11]. A single study has hardly identified all the microbes related to the disease due to the difference in analysis approaches, sampling methods, and participant demographic information. Integrating public knowledge to identify consistencies across studies through a meta-analysis has been employed to determine the intestinal microbiota, which allows researchers to solve false positives and negatives in the microbiome [12].

To explore the consistent biomarkers between superficial gastritis (SG) and AG, we collected seven published articles about SG and AG with 16S rRNA sequencing of gastric microbiome data, of which 367 samples were subjected to the 16S rRNA pipeline to reprocess data for systematic analysis. An integrated analysis was conducted to determine the potential biomarkers for gastritis, thereby reducing the occurrence of gastric cancer in the root. Additionally, we locally collected 78 gastric biopsy tissue samples from SG and AG subjects to confirm the effects of the identified microbes on gastritis development. Tools of reverse ecology utilize microbial genomic information to reconstruct metabolic networks, enabling the identification of exogenous metabolites. This approach offers us a strategy for identifying microbiota-targeted foods [13]. Based on the identified microbes, we inferred exogenous nutrients and recommended bacteria-targeted foods through the approach of reverse ecology. Our study, for the first time, identifies the biomarkers of global AG with or without *H. pylori* infection and puts forward a recommended diet according to the biomarkers, which can provide a preliminary basis for microbiome-directed prevention and therapy.

## 2. Materials and Methods

### 2.1. Publicly Available Metagenomic and 16S rRNA Datasets

We identified studies on the human gastric microbiome using keywords, including gastric microbiota, gastric microbiome, gastric microbes, gastric microflora, stomach microbiota, stomach microbiome, stomach microbes, stomach microflora, and gastritis (healthy control (HC), SG, and AG) in PubMed (419) and Google Scholar (manual search) until January 2023. Patients aged > 18 years who were provided gastric antrum tissue, body, or stomach fluid were preliminarily considered worldwide. Cohorts that did not provide data and did not mention gastritis were excluded. Studies with publicly available raw 16S rRNA data covering samples from six countries were included (Table 1).

### 2.2. Experimental Settings for 16S rRNA Datasets

Gastric 16S rRNA sequences from 12 publicly available studies were downloaded and imported into a pipeline, where quality control was conducted according to the first base, with a quality score Q  <  20. Data reads with quality scores of less than 20 were discarded. We filtered out operational taxonomic units (ASVs) with < 0.1% of the data, and the sequencing depth was limited to 4000 per sample.

### 2.3. Subject Recruitment and Sample Collection

A total of 100 male and female volunteers and patients (18 years of age or older) were invited to the affiliated hospital of Jiangnan university between August 2022 and January 2023. Subjects who had received proton pump inhibitors, antibiotics, or probiotic treatment in the past three months as well as those with other gastric diseases were excluded. Primary screening was based on the 13C-urea breath test to identify individuals with or without *H. pylori* infection. All patients with SG or AG were invited to participate in our experiment during their examination in the outpatient unit of the same department and were confirmed as having SG or AG using gastroscopy according to the chronic gastritis guidelines built in China [14]. Fresh gastric antrum tissue was obtained from each participant during standard esophagogastroscopy by an experienced physician. The participants were required to record information about their age, sex, and history of antibiotic use. Informed consent was obtained from all participants prior to gastroscopy or sample processing. Ethical approval of this study was approved by the Jiangnan University Human Research Ethics Committee (JNU20220606IRB05) and recorded by the Chinese Clinical Trial Registry (ChiCTR2200062661).

### 2.4. DNA Extraction of Gastric Antrum Tissue and MiSeq Sequencing

The gastric mucosal samples were gathered during endoscopy, frozen immediately at −80 °C, and prepared for 16S rRNA sequencing. DNA from the antral gastric samples was extracted and purified using the QIAamp DNA Mini Kit (Qiagen, Valencia, CA, USA). The bacterial 16S rDNA V3-V4 region was amplified using V3-V4 specific primers (341F, 5′-CCTACGGGNBGCASCAG-3′; 805R, 5′-GACTACNVGGGTATCTAAT CC-3′). After purification, the products were then paired-end (150 × 150 bp) sequenced on an Illumina Hiseq 2500 PE250 platform. The raw Illumina read sequencing data were stored in the National Center for Biotechnology Information database under the accession number PRJNA932133.

### 2.5. ASV Construction and Taxonomic Assignment

An alpha diversity analysis of the ASVs was conducted using the R package version 3.4.0, as shown by the ACE indices. Principal component analysis and principal coordinates analysis were generated based on the UniFrac distance to show the dissimilarities in the microbiome space between the group samples. Before the ASV analysis, we adjusted the data by age and sex using multivariate logistic regression. An abundance comparison of taxa between SG and AG was conducted using linear discriminant analysis effect size (LEfSe). *p*-values were calculated using the Wilcoxon test for differences between the two groups, and significant alterations were selected according to linear discriminant analysis (LDA) scores larger than 2.0.

### 2.6. Adopted Machine Learning Methods

A random forest identified microbes with the highest predictive ability because of intrinsic feature selection. The model was employed using the Random Forest (v4.6.12) R package, followed by 1000 random seeds. To precisely identify the core set of microbial markers, we randomly selected reanalyzed 16S rRNA data as training data. The random selection was processed ten times, and the validation area under the curve (AUC) (R 3.3.0, pROC package) was used to calculate the largest accuracy for the classifier. Furthermore, we used a ten-fold cross-validation to evaluate the classifier accuracy of the discovery cohort.

### 2.7. Prediction of Metagenomic Functions

The PICRUSt (v2.0.0) pipeline with default parameters was employed to predict the metabolic potential of microbiota according to data from the Human Microbiome Project using the ancestral state reconstruction algorithm. Differential functional orthologs and pathways were selected through a paired *t*-test, and *q* values < 0.05 were considered as significantly enriched.

### 2.8. Exogenous Metabolites and Whole Foods Prediction

We downloaded the genomes, proteomes, and KEGG orthology annotation files representing the standard strains of the entire genus from the IMG server. The selection of strains is based on commonly found bacteria within their genera. Metabolic models of these bacteria were reconstructed using RevEcoR (v0.99.3). As of now, the gastric microbiota has not been extensively characterized, and there is limited research on the common strains of gastric microbiota [15]. Furthermore, based on netseed, the exogenous nutritional requirements of these bacteria were predicted. The Canadian Food Database (FooDB, https://foodb.ca/, accessed on 7 August 2023) was further used to explore rational dietary interventions based on exogenous metabolites. Based on these exogenous metabolites, we identified a large number of diet options and summarized them in a list. We manually filtered out unquantified diets, as well as processed foods, alcohol, and those products without a clearly defined composition. Foods with high levels of exogenous metabolites and broad regulatory capabilities on microbiota were selected.

### 2.9. Statistical Tests

The LEfSe and random forest models were used to evaluate species with significant alterations. Multivariate linear regression was used to solve the problems of univariate statistics using the limma R package (v3.56.0), and factors including age and sex were incorporated into the analysis. Statistical analyses were performed using Origin software 2020 (Origin Lab Corp. v9.7.0.185). Corrected *p*-values < 0.05 differentially identified the taxa.

## 3. Results

### 3.1. Published 16S rRNA Data of Gastritis Included in This Study

To investigate the reproducible association between the gastric microbiome and AG, 16S rRNA data of the gastric mucosa of 537 samples were gathered from 12 different publicly available studies, including controls and patients with AG and SG. The 14 sample locations were Qingdao (China), Hanyang (Korea), Michigan (USA), Porto (Portugal), Inner Mongolia (China), Tumaco (Columbia), Túquerres (Columbia), and Seoul (Korea) (Table 1) [8,16,17,18,19,20,21,22,23,24,25,26,27]. We reran the datasets on our platform (Jiangnan University) to obtain a uniform result. After preprocessing and quality control, data from Qingdao (China), Beijing (China), and part of Hanyang (Korea) were excluded from the high-quality sequencing collection [17,22,25]. In addition, it was not possible to determine whether gastric fluid could be analyzed together with samples from the gastric antrum; therefore, we discarded the samples of gastric fluid [18,24] (Figure S1).

**Figure 1:**
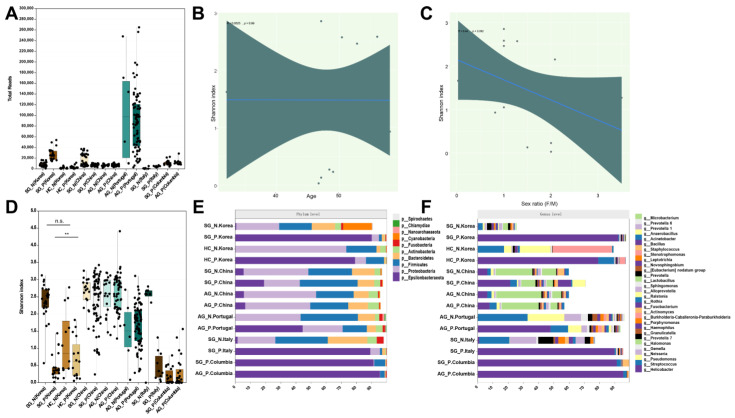
Bacterial characteristics and covariate analysis among selected studies. (**A**) Boxplots reporting the total number of reads in each dataset. (**B**,**C**) Multivariate analysis of species richness using crude age- and sex-adjusted coefficients obtained from linear models. Deep green represents the confidence interval and blue represents the regression line. (**D**) Shannon index of each group across different geographical locations. (**E**,**F**) Stacked bar plot shows the mean relative proportions of gastric microbial communities at the phylum and genus levels. HC_N, SG_N, and AG_N are representative of people without *H. pylori* infection; HC_P, SG_P, and AG_P are representative of people with *H. pylori* infection. n.s. represents no significance; **: *p* < 0.01. Circles in (**A**–**D**) represent each subject.

The effect of study-associated heterogeneity was investigated because of technical and biological differences in each study. (Figure 1A). The selected studies were read more than 5000 times, which suggested that they were of high quality. We compared the selected studies with available factors, including sex and age, and found no significant differences (Figure 1B,C). We investigated whether location leads to differences in microbiota diversity, and the countries showed statistically significant differences. We found that the microbiota diversity between HC_N and SG_N was similar, while there was a difference between HC_P and SG_P (Figure 1D). Furthermore, we determined the diversity and composition of the gastric microbiota that were enriched or depleted in the SG and AG groups consistently across the six populations in different locations. Six selected studies did not demonstrate the situation of *H. pylori* infection in their samples; thus, we considered *H. pylori* with a relative abundance of more than 1% as *H. pylori* infection [16]. The results exhibited a similar trend in that *H. pylori* infection in patients with SG led to a decrease in the Shannon index, and the infection exhibited an increase in the index in the AG group across all studies (Figure 1F). The gastric bacterial composition at the phylum and genus levels differed. Individuals with *H. pylori* infection had a high proportion of Epsilonbacteraeota, whereas high proportions of Proteobacteria, Firmicutes, and Bacteroidetes were observed in individuals without the infection (Figure 1E). At the genus level, bacteria in the *H. pylori* infection group, except in China, were dominated by *Helicobacter* and *Streptococcus*, while the gastric bacteria of the Chinese group were occupied by *Helicobacter*, *Streptococcus*, *Halomonas*, and *Lactobacillus* (Figure 1F). Across all the studies, although people in different places have various bacterial compositions, *H. pylori* infection drives the bacteria to a consistent composition. Studies have shown no differences between HC_N and SG_N. Patients with AG were different from those in the HC and SG groups, showing greater bacterial diversity.

### 3.2. Gastric Microbial Diversity of CKD between SG and AG

**Figure 2:**
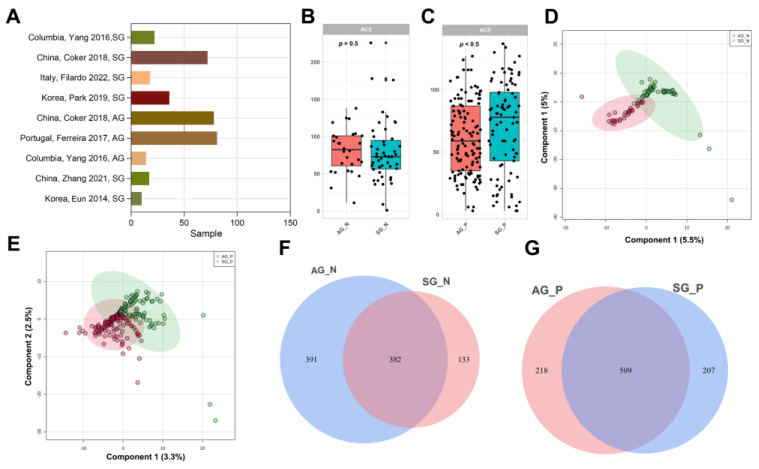
Population and gastric bacteria diversity across SG and AG cohorts. (**A**) Sample sizes of SG and AG populations. The ACE species diversity between patients with SG and AG with (**B**) or without *H. pylori* infection (**C**). Circles in (**B**) and (**C**) represent each subject. The PLSDA based on ASV distribution between patients with SG and AG with (**D**) or without *H. pylori* infection (**E**). Venn diagrams displaying the overlaps between groups with (**F**) or without *H. pylori* infection (**G**). Analyses are performed at the genus level. SG_N (*n* = 63), AG_P (*n* = 42), SG_P (*n* = 111), AG_P (*n* = 128) [3,10,13,14,16,17,20].

To investigate the global biomarkers between SG and AG with or without *H. pylori* infection, as well as *H. pylori*-assistant bacteria, we included seven suitable datasets for investigation (Figure 2A). During our analysis of SG and AG biomarkers, we divided the patients into *H. pylori* infection and non-*H. pylori* infection. Because patients with 100% pylorus infection were not conducive to the observation of biomarkers, we excluded them (SG191, SG193, SG194, SG195, SG203, SG198, AG161, AG162, AG163, AG164, AG169, AG170, and AG171). When all datasets were aggregated, a significant ACE change in the microbiota was found in patients with *H. pylori* infection (*p* < 0.05). To investigate the association between the functional composition of the gastric microbiota and disease, we quantified the Bray–Curtis dissimilarity between all datasets. Our results showed that there was some overlay and separation, and the circle of the SG group trended toward that of the AG group (Figure 2D,E). Venn diagrams indicated that 382 ASVs were shared between AG_N and SG_N, and 509 ASVs were shared between AG_P and SG_P.

### 3.3. Gastric Microbial Biomarkers Related to AG

**Figure 3:**
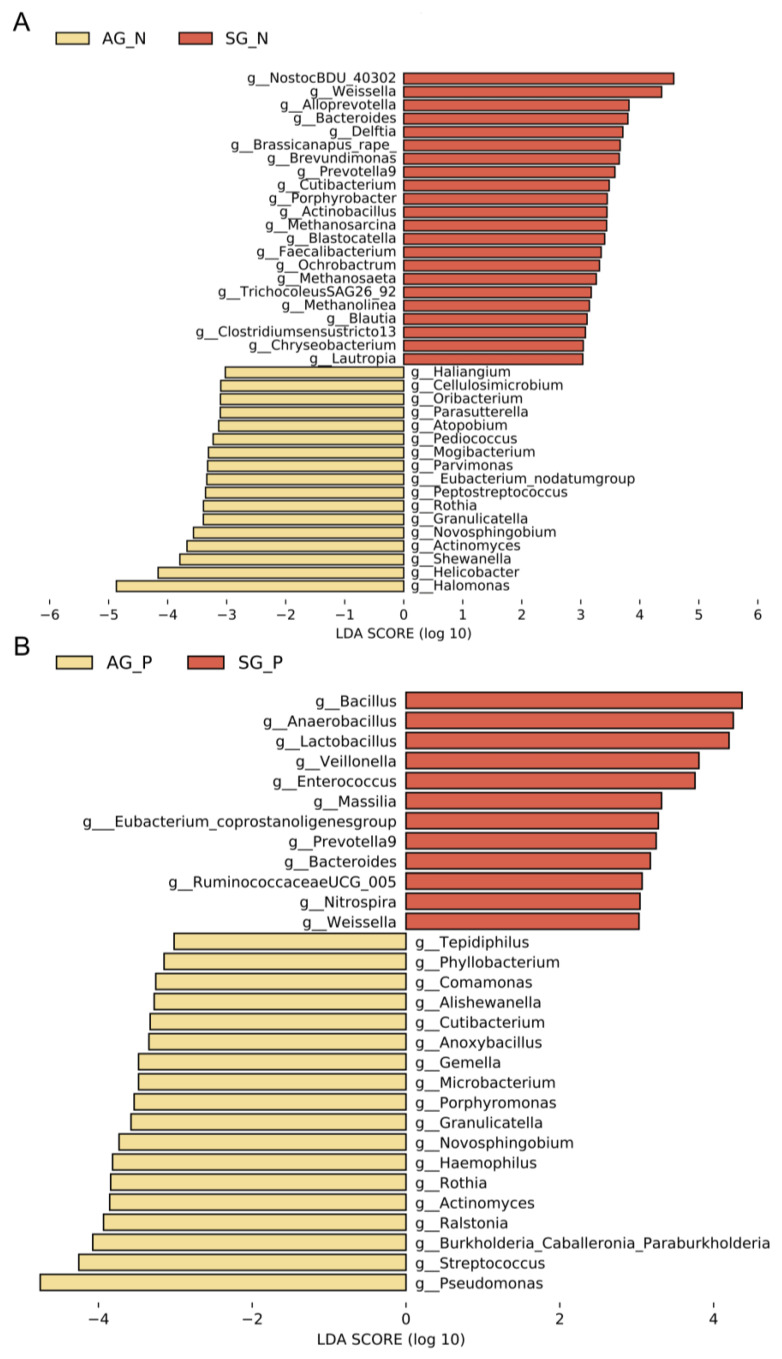
LEfSe analysis of gastric bacteria. Histograms of differential species between SG and AG. (**A**,**B**) SG-enriched species are indicated with a negative LDA score (red), and species enriched in the AG group are indicated with a positive LDA score (yellow). Only species with an LDA score  > 3 at *p* < 0.05 are shown. SG_N (*n* = 63), AG_P (*n* = 42), SG_P (*n* = 111), AG_P (*n* = 128).

LEfSe was used to differentiate between the specific microbiota of SG and AG based on operational taxonomic units. The results showed that 17 bacteria, including *Halomonas*, *Helicobacter*, and *Shewanella* were predominant in AG_N, whereas 22 bacteria, including *Weissella*, *Alloprevotella*, and *Bacteroides* were reduced in AG_N compared to SG_N (Figure 3A). When we analyzed the biomarkers for SG_P and AG_P, we excluded *H. pylori,* as a large proportion of the bacteria interfered with our investigation. (Figure 3B). In addition, 12 bacteria, including *Bacillus*, *Anaerobacillus*, and *Lactobacillus*, were enriched in SG_P, compared to 18 bacteria, including *Streptococcus* and *Pseudomonas,* in AG_P.

### 3.4. Repeatable Gastric Microbial Markers for AG

**Figure 4:**
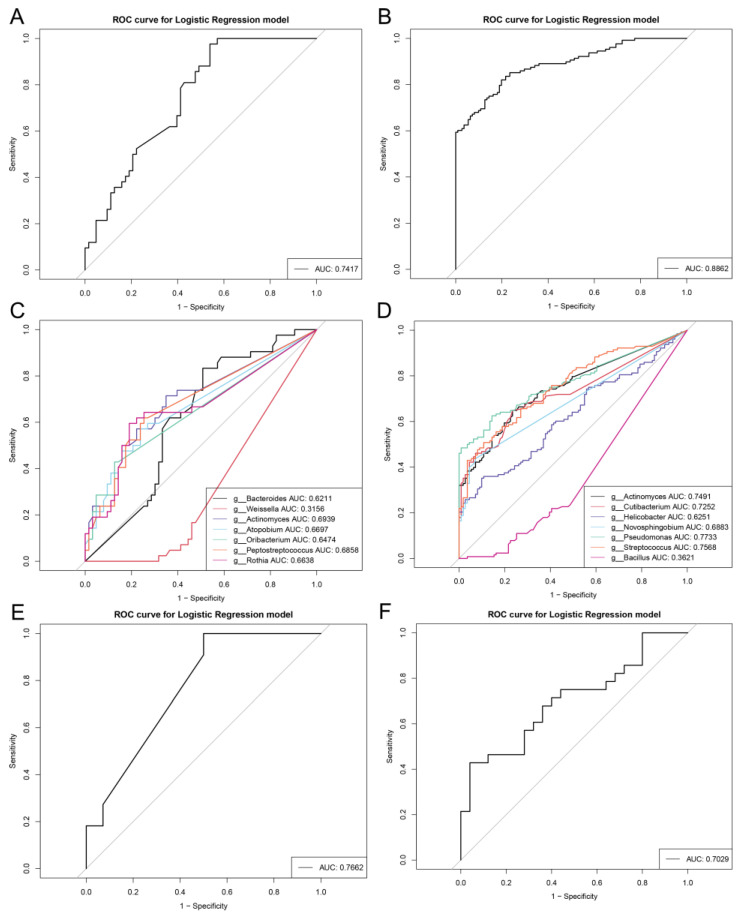
Random forest classifier model based on crucial bacteria. In the discovery cohort, seven gastric biomarkers between SG_N (*n* = 63) and AG_N (*n* = 42) are selected as the important microbiota (AUC of 0.7417) (**A**,**C**). Seven gastric biomarkers between SG_P (*n* = 111) and AG_P (*n* = 128) are selected as the important microbiota (AUC of 0.8862) (**B**,**D**). In the validation cohort, the AUCs of predictive models achieve 0.7662 between SG_N (*n* = 14) and AG_N (*n* = 11) and 0.7029 between SG_P (*n* = 28) and AG_P (*n* = 25), respectively (**E**,**F**).

Furthermore, a random forest classifier model between the SG and AG groups was constructed to explore the potential of repeatable gastric microbial markers. Microbial markers were identified according to the results of the LEfSe, random forest scores, and *p*-values (Table S1). Seven ASVs, *Bacteroides*, *Weissella*, *Actinomyces*, *Atopobium*, *Oribacterium*, *Peptostreptococcus*, and *Rothia,* were selected as biomarkers between SG_N and AG_N to build a ten-fold cross-validation of the random forest model (Figure 4C). Seven ASVs had an AUC of 0.7417 between SG_N and AG_N (Figure 4A). We employed a cluster-to-random forest machine to diagnose AG_P based on the microbial abundance. Among the clusters, *Bacillus* was found to be decreased in AG_P, while microbes, including *Actinomyces*, *Cutibacterium*, *Helicobacter*, *Novosphingobium*, *Pseudomonas*, and *Streptococcus*, were enriched compared to SG_P (Figure 4D). Simultaneously, a random forest classifier based on the signatures of SG_P and AG_P obtained reasonable results, reaching an AUC of 0.8862 (Figure 4B). The data indicated that the classifier model based on biomarkers had powerful diagnostic potential for differentiating AG from SG. To explore the precision of these gastric microbiota as classifiers, we randomly recruited participants with SG or AG with or without *H. pylori* infection for validation according to a sophisticated doctor’s diagnosis in China. In the validation cohort, the model between SG_N and AG_N showed higher AUC values of 0.7662, whereas the classifier between SG_P and AG_P produced an AUC of 0.7029, compared with the AUC used in the discovery cohort.

### 3.5. Crucial Bacteria and Microbial Functions Related to AG

**Figure 5:**
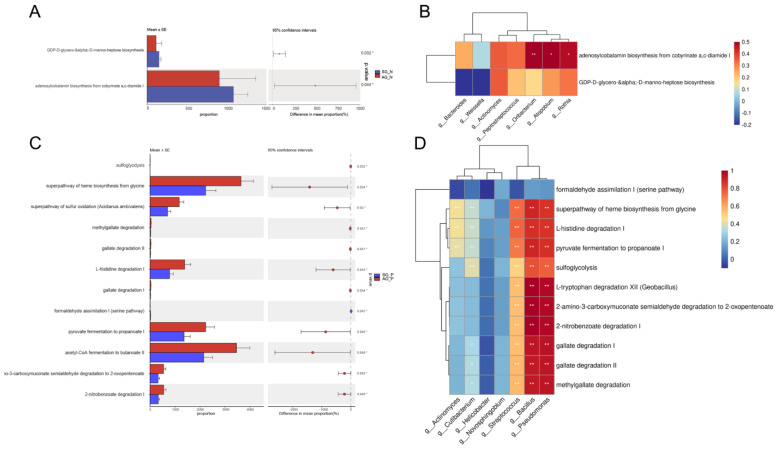
Microbiota-related predicted functions between SG and AG. Predicted functions of AG-associated gastric microbes with significant changes based on the validation cohort (**A**,**C**). Two predicted microbial functions are significantly decreased in AG_N (*n* = 11) compared to SG_N (*n* = 14) (**A**). Ten predicted microbial functions were increased in AG_P (*n* = 25) compared to SG_P (*n* = 28) (**C**). Heatmaps indicate the correlation between different gut microbes and pathways (**B**,**D**). *: *p* < 0.05; **: *p* < 0.01.

The Kyoto Encyclopedia of Genes and Genomes (KEGG) pathway profiles were determined using the PICRUSt2 pipeline. Two predicted functions were decreased in the AG_N group compared with the SG_N group, except for the adenosylcobalamin and GDP-D-glycero-α-D-manno-heptose biosynthesis pathways (Figure 5A). Parameters including sulfoglycolysis, the superpathway of heme biosynthesis, methylgallate degradation, gallate degradation II, gallate degradation I, L-histidine degradation, formaldehyde assimilation I (serine pathway) I, 2-amino-3-carboxymuconate semialdehyde degradation to 2-oxopentenoate, 2-nitrobenzoate degradation, pyruvate fermentation to propanoate, and L-tryptophan degradation XII (Geobacillus) were higher in AG_P, while formaldehyde assimilation I (serine pathway) decreased in AG_P compared with SG_P (Figure 5C). Furthermore, Spearman correlation coefficients were calculated between key microbes and pathways. The abundances of *Oribacterium*, *Atpobium*, and *Rothia* had positive relationships with adenosylcobalamin (Figure 5B). Gallate and amino acid degradation and heme biosynthesis had significant negative relationships with the abundances of *Actinomyces* and *Cutibacterium,* and had positive relationships with that of *Streptococcus*, *Bacillus*, and *Pseudomonas* (Figure 5D).

### 3.6. Dietary-Based Interventions for AG

To further characterize the diet targeting these missing microbial communities, we selected 18 representative strains capable of reflecting *Bacteroides* and *Weissella* based on the human gut microbiome gene catalog (Table S2) [28]. In order to infer the nutrients for these strains, we reconstructed the metabolic networks of these 18 strains using the RevEcoR software (v 0.99.3) and identified exogenous metabolites for these strains through the netseed software (Table S2) [13,29]. We discovered 104 exogenous metabolites between *Bacteroides* and *Weissella* and found 19 unique exogenous metabolites in *Weissella* compared to *Bacteroides*. Meanwhile, *Bacteroides* exhibited significantly stronger nutritional requirements than *Weissella*. Exogenous metabolites provide dietary information for targeting specific bacteria. We inquired about potential targeted diets using FooDB, the world’s largest metabolite–food network on the website. The compounds were detected in 708 foods (Table S3). Further, we manually excluded processed foods, alcoholic beverages, and some undefined items. In order to screen for the best microbiota-targeted foods, we finally selected foods with clearly defined quantification. The quantitative limits for cysteine, alpha-Diamino-beta-dithiolactic acid, N-methyltyramine, glutamic acid, methionine S-oxide, and raffinose were explicitly indicated in the entire range of foods. Therefore, based on the concentrations of these several exogenous metabolites, we ranked the foods (Table S4). Due to methionine S-oxide and raffinose being unique metabolites found only in *Weissella*, they had to be considered. Lastly, we selected soybean, common wheat, dates, and barley as potential dietary candidates for targeted microbial populations.

## 4. Discussion

Reverse ecology tools provide a promising bridge for studying the interaction between diet and bacteria [13]. With the increasing number of tools for studying bacterial metabolic models, genome-scale metabolic models are expected to become practical tools for personalized nutrition. Here, we have constructed dietary prediction methods by identifying microbiota biomarkers, exogenous metabolites, and whole foods.

Firstly, we collected 12 publicly available datasets from different regions and comprehensively evaluated the AG-related gastric microbiome and the microbial ability to distinguish newly diagnosed patients with AG from those with SG. In these studies, different sampling methods were employed to examine the gastric microbiome, including gastric washes and endoscopic sampling. Some studies have used gastric fluid to consider the microbiome, and we confirmed that the microbiome in the gastric fluid and gastric body is different, and the combination of gastric fluid and body samples might be unreasonable, which is similar to the results of Sung et al. [27,30] (Figure S1).

In addition, samples of gastric mucosa from healthy individuals have been identified as having superficial gastritis. Therefore, we explored the differences between normal controls and patients with superficial gastritis in terms of microbial composition and diversity. Surprisingly, different microbial diversity was found in SG_P compared to HC_P, while significant changes were not found in SG_N compared to HC_N, which suggests that *H. pylori* is a crucial driving factor, and SG_P and HC_P cannot be diagnosed together (Figure 1D–F). We also evaluated the variability across the gastric microbiome. Microbial diversity in the available datasets was independent of potential confounding factors such as age and sex, while dissimilarity in geographical regions was observed, which is similar to He et al. [31] (Figure 1B,C).

We selected 376 16S rRNA samples, including only SG and AG, and explored the differences between the SG and AG microbial communities (Figure 2). We found a shift in the microbial community from SG to AG according to the principal coordinates analysis, which suggested a continuous change regardless of other factors. The abundance of *H. pylori* was excluded when we explored microbial markers between SG_P and AG_P, since microbes with large portions could easily mask the signals of other microbes [32]. LEfSe and random forest scores are the most widely used identification methods for distinguishing biomarkers in microbial studies [33]. Finally, seven microbial markers for AG_N and AG_P were determined using LEfSe and random forest scores, respectively. More importantly, the microbes predicted AG detection accuracies of 0.7662 and 0.7029 for AG_N and AG_P in our local cohorts, respectively, which suggests repeatable biomarkers.

Most of the AG biomarkers explored in this study were enriched in AG, including *Bacteroides*, *Actinomyces*, and *Peptostreptococcus* for AG_N, and *Actinomyces*, *Pseudomonas*, and *Streptococcus* for AG_P. As expected, some potential synergistic bacteria of *Helicobacter* promoted the development of AG_P (Figure 4C,D). These identified microbes are in agreement with previous studies and may develop into intestinal metaplasia and gastric cancer without prevention [6,7,16,22,23,30,34]. We identified *Actinomyces* as a biomarker for both AG_N and AG_P. Microbes are filamentous Gram-positive *Bacillus* that weaken the host immune system or decrease oxygen tension and are strongly associated with inflammation [35]. Furthermore, AG_N-associated beneficial bacteria, *Weissella* and *Bacteroides*, produce bacteriocins to inhibit pathogens and ameliorate inflammation, which can be considered as a protective role for AG_N [36,37]. *Peptostreptococcus* possessing lactic acid and *Atopobium* producing H2S are acid-forming bacteria that are not conducive to the balance of stomach acid and can be used as indicators of AG_N progression [38,39]. However, the number of harmful bacteria was significantly increased in patients with AG_P. *Streptococcus* is widely regarded as an AG_P-associated bacterium, and species including *Streptococcus* mitis/oralis, *S. pneumoniae*, *S. anginosus*, and *S. salivarius* are reportedly associated with AG in *H. pylori*-positive subjects [16,23,30]. *Cutibacterium* and *Novosphingobium* are related to disease progression, but the pathogenic mechanism remains unknown, and the health association of Pseudomonas remains debatable [40,41,42].

The metabolic function of gastric microbes is one of the factors affecting disease development. Some predicted metabolic shifts between SG and AG were discovered (Figure 5). Adenosylcobalamin and GDP-D-glycero-α-D-manno-heptose biosynthesis are the two main pathways that are depleted in AG_N. Adenosylcobalamin is known as a member of vitamin B12, whose deficiency is often found in patients with gastritis. A reduction in vitamin B12 biosynthesis in microbiota in AG_N further promotes vitamin B12 deficiency, inducing high disease risk, while the biosynthesis is potentially related to Oribacterium, Atpobium, and Rothia [43,44,45]. The GDP-D-glycero-α-D-manno-heptose biosynthesis in SG_N remained unclear, while heptose-containing bacterial natural products are considered sources of novel bacterial drugs [46]. In patients with *H. pylori* and AG infections, formaldehyde assimilation I (serine pathway) was the only pathway that showed a significant decrease [47]. Formaldehyde assimilation is a pathway for bacterial detoxification that plays important roles in methylotrophs, and depletion of this pathway may lead to a decrease in *Bacillus* [48]. The enrichment of heme biosynthesis has been reported to promote bacteria in Proteobacteria and inflammation in the gut [49,50]. Gallates have excellent bioactivity for bacterial inhibition and a reduction in inflammation [51]. Our observation of gallate degradation in AG_P suggests that a reduction in gallate levels in the stomach may induce an increase in AG_P-related microbes. An increase in pathways including histidine degradation, pyruvate fermentation to propanoate, and L-tryptophan degradation has been previously found in patients with gastritis [25]. Therefore, our findings indicate that vitamin B12 biosynthesis can be an essential pathway to distinguish AG_N and SG_N, whereas the pathogenic mechanism of AG_P is mainly related to gallate and amino acid degradation and heme biosynthesis. However, the roles of these significantly altered functional pathways in the development and occurrence of AG require further study [47].

The supplementation of beneficial microbes has shown potential in alleviating diseases. However, due to various factors such as a lack of approval or colonization difficulties, direct bacterial supplementation is often restricted [47]. Recently, there is growing interest in using microbiota-directed diets to modulate the microbes. Considering the unknown safety of *Bacillus*, which shows reduced abundance in AG_P patients, it is recommended to supplement gallate directly to inhibit harmful bacteria. Gallate has been reported to have the ability to suppress *H. pylori* [48]. For AG_N, two crucial genera, *Weissella* and *Bacteroides*, have been identified. Dead bacteria do not possess colonization capability and are easily washed away by digestive fluids. Our samples were taken from gastric antral tissue, ensuring a certain level of bacterial viability. On the other hand, bacterial transcripts can identify active bacteria. Wurm et al. analyzed the transcripts of gastric microbiota and found a high proportion of *Bacteroides* [49]. Furthermore, Mailhe et al. also isolated various *Bacteroides* from the stomach [50]. *Weissella*, due to its low pH tolerance, also has the ability to survive in the stomach [51,52]. All of the above ensures that these bacteria can survive in the stomach and benefit from potential prebiotics. Key strains from these genera were selected to represent the entire genus. A reverse ecology approach was applied for reconstruction of models and the identification of exogenous metabolites associated with these strains [13]. Based on the FooDB database, soybean, common wheat, dates, and barley have been recognized as key foods that may alleviate AG_N. Due to the predictive nature of adenosyloobalamin-related foods in the FooDB database, we have not included foods related to adenosyloobalamin. However, it is recommended to combine these bacteria-targeted foods with adenosyloobalamin-rich sources to maximize therapeutic effects.

## 5. Conclusions

In conclusion, our study provides microbial biomarkers of AG and a recommended diet through a reverse ecology approach. We expect that our results from the collected cohort will provide direction for the further investigation of AG. However, there are still limitations. The meta-analysis could not determine interfering factors, such as sample handling, or cohort information, such as geography, medical background, and diet. Next, the staging of gastritis is clinically helpful for monitoring the progression of the disease. However, due to the lack of research on the microbiota of different degrees of gastritis in collected datasets, our sample classification was not stratified according to gastritis staging. The availability of accessible metagenomic data from the stomach and the detailed composition of gastric microbiota at the species level are limited, which may result in inaccuracies in the predicted results. Nevertheless, this study still provides a set of dietary strategies that can be followed, focusing on microbiota.

## Figures and Tables

**Table 1:** Cohort details of the studies included in the meta-analysis. Continuous variables were expressed as means ± standard deviations (s.d.).

Reference	Dataset Geographic Regions (Data Source)	Sequence Accession Codes	Samples Used in This Study	Age (Average ± s.d.)	Sex F(%)/M(%)
Wang et al., 2016 [12]	Qingdao (China)	SRP060550	SG (6)	55.8 ± 13.5	39.7/60.3
Eun et al., 2014 [13]	Hanyang (Korea)	SRP038955	SG (10)	50.4 ± 11.5	50/50
Zhang et al., 2021 [14]	Nanjing (China)	PRJNA634837	SG (17)	56 ± 10.6	47.1/52.9
Seekatz et al., 2019 [15]	Michigan (USA)	PRJNA495320	HC (49)	37.7 ± 10.2	34.7/65.3
Ferreira et al., 2017 [3]	Porto (Portugal)	PRJNA413125	AG (81)	43.6 ± 7	2.5/97.5
Park et al., 2019 [16]	Hanyang (Korea)	PRJNA389357	SG (62)	32.1 ± 10.5	58.1/41.9
Coker et al., 2018 [10]	Inner Mongolia (China)	PRJNA375772	SG (52)	52.8 ± 15	50/50
			AG (62)	56 ± 12.3	50/50
Coker et al., 2018 [10]	Xian (China)	PRJNA375772	SG (20)	47.1 ± 11.7	50/50
			AG (16)	50.4 ± 11.7	56.2/43.8
Filardo et al., 2022 [17]	Rome (Italy)	PRJNA795512	SG (18)	50.4 ± 11	77.8/22.2
Bassis et al., 2015 [18]	Michigan (USA)	PRJNA263948	HC (34)	41.3 ± 16.2	67.6/32.4
Wang et al., 2020 [19]	Beijing (China)	PRJEB26931	HC (51)	44.8 ± 13.6	53/47
Yang et al., 2016 [20]	Tumaco (Columbia)	PRJEB11763	SG (12)	49 ± 6.7	66.7/33.3
			AG (6)	46.7 ± 2.1	66.7/33.3
Yang et al., 2016 [20]	Túquerres (Columbia)	PRJEB11763	SG (10)	47.1 ± 4.4	60/40
			AG (8)	48.4 ± 7.5	75/25
Sung et al., 2016 [21]	Seoul (Korea)	GSE61493	HC (31)	58 ± 11.6	44.9/55.1

## Data Availability

The datasets supporting the conclusions of this article are available in the National Center for Biotechnology Information repository under the accession number PRJNA932133.

## Supplementary Materials

The following supporting information can be downloaded at https://www.mdpi.com/article/10.3390/nu15224738/s1: Figure S1: α-diversity of microbiota between healthy people of gastric antrum (*n* = 29) and gastric fluid (*n* = 84); Table S1: Lists of microbial biomarkers between SG and AG; Table S2: Lists of exogenous compound of 18 representative strains in Bacteroides and Weissella; Table S3: Lists of food related to exogenous compound; Table S4: Lists of quantification of exogenous compound in food.
