# Supplementary figures and images for: Predicting Personalized Diets Based on Microbial Characteristics between Patients with Superficial Gastritis and Atrophic Gastritis
Source: Nutrients. 2023 Nov 9;15(22):4738. doi: 10.3390/nu15224738 (PMC10675729; doi:10.3390/nu15224738)

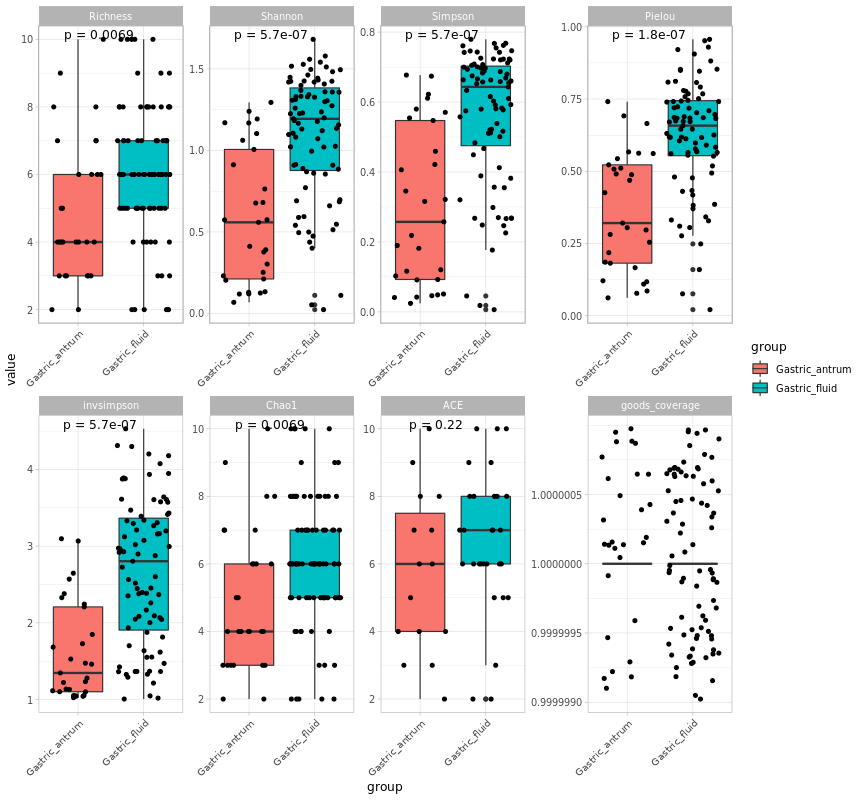

Supplement: Supplementary file 1 [file nutrients-15-04738-s001.zip › figureS1.png]
